# Draft Whole-Genome Sequences of Three Diarrheagenic Escherichia coli Strains Isolated from Farmed Deer in New Zealand

**DOI:** 10.1128/genomeA.00300-18

**Published:** 2018-04-19

**Authors:** A. Springer Browne, Patrick J. Biggs, Alice Elliott, Patricia Jaros, Nigel P. French, Anne C. Midwinter

**Affiliations:** a ^*m*^EpiLab, Infectious Disease Research Centre, School of Veterinary Science, Massey University, Palmerston North, New Zealand; b Institute of Fundamental Sciences, Massey University, Palmerston North, New Zealand

## Abstract

Escherichia coli bacteria commonly colonize the gastrointestinal tracts of farmed ruminants. Cattle are a well-recognized reservoir of zoonotic E. coli; we report here, however, the draft genome sequences of three diarrheagenic E. coli strains isolated from farmed red deer (Cervus elaphus) in the Manawatu region of New Zealand.

## GENOME ANNOUNCEMENT

Diarrheagenic Escherichia coli strains may cause a wide spectrum of human disease outcomes, ranging from significant clinical consequences such as hemolytic uremic syndrome (Shiga toxin-producing E. coli [STEC]) to mild watery diarrhea (enteropathogenic E. coli [EPEC]) (1, 2). Ruminants are a reservoir of zoonotic STEC and EPEC strains, and “contact with animal manure” and “living close to cattle” were identified during a recent case control study as risk factors for human STEC disease in New Zealand (3). The feces of farmed red deer (Cervus elaphus) contain high concentrations of E. coli (4), and epidemiological data have demonstrated deer or venison as a likely source of infection in several outbreaks of human STEC disease (5, 6).

The use of animals and sample collection for this study were approved by the Grasslands Animal Ethics Committee (Animal Ethics application 13237, Grasslands, Palmerston North, New Zealand). Rectoanal mucosal swabs were used to sample the feces of healthy red deer from two farms in the Manawatu region of New Zealand during 2014, and STEC O103:H2, STEC O157:H7, and EPEC O121:H19 strains were isolated. Genomic DNA of the three strains was isolated from a single colony using the QIAamp DNA minikit (Qiagen, Hilden, Germany), and in-house library preparations were made using the Nextera XT DNA library preparation kit (Illumina, San Diego, CA). Paired-end sequencing (2 × 125 bp) was performed using the Illumina HiSeq platform with the HiSeq reagent v4 kit (Illumina, San Diego, CA) at New Zealand Genomics Limited (University of Otago, Dunedin, New Zealand).

Raw reads were processed, assembled, and analyzed using the Nullarbor pipeline (v1.20) in the “accurate” mode (7). Serotypes of bacterial isolates were determined using SerotypeFinder (8). Draft genomes were annotated using the NCBI Prokaryotic Genome Annotation Pipeline. Genome sizes, number of contigs, and virulence genes are presented in Table 1.

**TABLE 1:** Description of E. coli strains sequenced, their genomic characteristics, and associated virulence factors

Strain	GenBank accession no.	Serotype^a^	Source farm	Length (bp)	No. of contigs	Fold coverage (×)	*N*_50_ (bp)	Multilocus sequence type^b^	Virulence genes^c^
DV112i	PELG00000000	O103:H2	B	5,448,699	299	66	119,377	ST-17	*cif*, *eae*, *ehxA*, *espA*, *espB*, *espI*, *espJ*, *etpD*, *gad*, *iss*, *nleA*, *nleB*, *nleC*, *stx1A*, *stx1B*, *tir*
DV60u	PELH00000000	O121:H19	A	5,246,870	185	107	182,793	ND^d^	*astA*, *eae*, *ehxA*, *espA*, *espB*, *espF*, *espJ*, *etpD*, *gad*, *iha*, *lpfA*, *nleA*, *nleB*, *tir*, *toxB*
DV42a	PELI00000000	O157:H7	A	5,312,372	237	114	182,793	ST-11	*astA*, *eae*, *ehxA*, *espA, espB*, *espJ*, *espP*, *etpD*, *gad*, *iha*, *iss*, *katP*, *nleA*, *nleB*, *nleC*, *stx2A*, *stx2B*, *tccP*, *tir*, *toxB*

a *In silico* serotype was determined using SerotypeFinder v1.1 (8).

b Multilocus sequence type was determined using Multilocus Sequence Typing (MLST) v1.8 (13).

c Virulence genes were identified using VirulenceFinder v1.5 (14).

d ND, not determined.

The O157:H7 serotype is commonly isolated from humans with clinical STEC infection; it was isolated from 88.1% (170 of 193) of all human cases where STEC was isolated in New Zealand in 2014 (9). The adoption of PCR-based methods for the detection of *stx* genes by several New Zealand District Health Boards during the later stages of 2016 has contributed to an increased detection of non-O157 STEC serogroups (46.9%, 181 of 386 of all cases where STEC was isolated) (10), including that of STEC O103:H2 (2 cases in 2016) (11). STEC O121:H19 strains are rarely found in New Zealand, although two were isolated from human cases of disease in 2013 (12). Enteropathogenic E. coli cases are not notifiable in New Zealand, and thus the burden of human disease associated with *stx*-negative pathotypes is unknown.

Epidemiological studies and genome sequence data from STEC and EPEC strains will provide important information to understand the role of farmed deer as a reservoir of zoonotic disease and will aid in future efforts to identify the modes of transmission between different ruminant species.

### Accession number(s).

The whole-genome shotgun sequences described here have been deposited in DDBJ/ENA/GenBank under the accession numbers listed in Table 1.
